# Trends in newborn umbilical cord care practices in Sokoto and Bauchi States of Nigeria: the where, who, how, what and the ubiquitous role of traditional birth attendants: a lot quality assurance sampling survey

**DOI:** 10.1186/s12884-017-1551-x

**Published:** 2017-11-09

**Authors:** Dele Abegunde, Nosa Orobaton, Katherine Beal, Amos Bassi, Moyosola Bamidele, Toyin Akomolafe, Francis Ohanyido, Olayinka Umar-Farouk, Saba’atu Danladi

**Affiliations:** 1 Targeted States High Impact Project (TSHIP), JSI Research & Training Institute, Inc., Bauchi, Bauchi State Nigeria; 2 JSI Research & Training Institute, Inc, Boston, USA; 3 Targeted States High Impact Project (TSHIP), JSI Research & Training Institute, Inc., Sokoto, Sokoto State Nigeria; 4 Targeted States High Impact Project (TSHIP), JSI Research & Training Institute, Inc., Abuja, Nigeria; 5 FUTURES- Targeted States High Impact Project (TSHIP), Bauchi, Bauchi State Nigeria

**Keywords:** Umbilical cord practices, Chlorhexidine, Traditional birth attendants, Nigeria

## Abstract

**Background:**

Neonatal infections caused by unsafe umbilical cord practices account for the majority of neonatal deaths in Nigeria. We examined the trends in umbilical cord care practices between 2012 and 2015 that coincided with the introduction of chlorhexidine digluconate 7.1% gel in Bauchi and Sokoto States.

**Methods:**

We obtained data from three rounds of lot quality assurance samples (LQAS) surveys conducted in 2012, 2013 and 2015. Households were randomly sampled in each round that totaled 1140 and 1311 households in Bauchi and Sokoto States respectively. Mothers responded to questions on cord care practices in the last delivery. Coverage estimates of practice indicators were obtained for each survey period. Local Government Area (LGA) estimates for each indicator were obtained with α ≤ 5%, and β ≤20% statistical errors and aggregated to State-level estimates with finite sample correction relative to the LGA population.

**Results:**

Over 75 and 80% of deliveries in Bauchi and Sokoto States respectively took place at home. The proportion of deliveries in public facilities reported by mothers ranged from 19% in 2012 to 22.4% in 2015 in Bauchi State and from 12.9 to 13.2% in 2015 in Sokoto State. Approximately 50% of deliveries in Bauchi and more than 80% in Sokoto States were assisted by traditional birth attendants (TBAs) or relatives and friends, with little change in the survey periods. In Bauchi and in Sokoto States, over 75% and over 80% of newborn cords were cut with razor blades underscoring the pervasive role of the TBAs in the immediate postpartum period. Use of chlorhexidine digluconate 7.1% gel for cord dressing significantly increased to the highest level in 2015 in both States. Health workers who attended deliveries in health facilities switched from methylated spirit to chlorhexidine. There were no observable changes in cord care practices among the TBAs.

**Conclusion:**

Unsafe umbilical cord care practices remained prevalent in Bauchi and Sokoto States of Nigeria, although a recent introduction of chlorhexidine digluconate 7.1% gel positively changed the cord care practices toward safer practices among public health providers. TBAs, friends and relatives played the strongest immediate postpartum roles and mostly retained the unsafe cord care practices such as use of ash, cow dung and hot compress. We recommend that existing TBAs are retrained and refocused to forge stronger links between communities and the primary health centers to increase mothers’ access to skilled birth attendants.

## Background

Globally, the persistent high rate of neonatal mortality particularly in many low income countries of Sub-Saharan Africa remains a major concern. In 2010 alone, over 3 million neonates were estimated to have died in 2010 worldwide [1]. Nigeria and India contributed the majority of the global burden of neonatal deaths [1–3]. There has been little improvement in the neonatal mortality rate (NMR) in Nigeria since the late 1990s. Nigeria’s neonatal mortality rates (NMR) were estimated at 37, 48, 41 and 37 per 1000 live births in 1999, 2003, 2008 and 2013 respectively [4]. Estimates by Akinyemi et al. are comparatively higher at 42, 49, 39 and 38 per 1000 live births in contiguous years [5]. They nonetheless suggest a pattern of lack of appreciable progress. The northeastern and northwestern regions of Nigeria fared the worst. For instance, NMR in Bauchi State in northeastern region were 33, 61, 53 and 43 per 1000 live births in 1999, 2003, 2008 and 2013 respectively [4]. Similarly, NMR estimates in the same years in Sokoto State in the northwestern region were 36, 55, 47 and 44 respectively [4]. A combined 36% of all causes of neonatal deaths were due to sepsis (21%) and tetanus (16%) – two complications of umbilical infections [4]. The proximate causes of neonatal infection were related to immediate postpartum cord-care choices and persisting negative cultural beliefs and practices.

Potentially harmful cord care cultural practices especially in relation to home deliveries predominates in parts of sub-Saharan Africa [6–8]. For instance, substances such as powders made of roots, burnt gourds or ash, petroleum jelly, commercial baby lotion, cooking oil and breast-milk were commonly applied to the umbilical cord [7]. Charcoal, baby powder, dust, lubricating agents (e.g. Vaseline, cooking oil, used motor oil), cow dung and chicken droppings [6] have been reported to have been applied to the umbilical cord in some cultural settings. About 87.5 and 79.3% of deliveries in the northwestern and northeastern regions of Nigeria took place at home including situations where no one was present with the mother at the delivery [9, 10]. Also, 80.1 and 87.7% of these deliveries in northwestern and northeastern regions are not assisted by a skilled health worker [11].

Between 2009 and 2015, United States Agency for International Development (USAID) funded the Targeted States High Impact Project (TSHIP)—managed by a consortium led by JSI Research and Training Institute, Inc., supported the governments of Bauchi and Sokoto States to reduce preventable maternal, newborn, infant, and child morbidity and mortality. The project components comprised of integrated maternal, newborn and child health (MNCH) with family planning (FP) and reproductive health (RH) interventions. The goal of the newborn component of this project included the reduction of neonatal mortality through increasing the mothers’ access to chlorhexidine digluconate 7.1% gel for newborn cord application to reduce cord infection, and a helping babies breathe program, to reduce mortality from birth asphyxia. The World Health Organization (WHO) had in 2013 recommended the application of daily chlorhexidine (7.1% chlorhexidine digluconate aqueous solution or gel, delivering 4% chlorhexidine) to the umbilical cord stump during the first week of life for newborns delivered at home in settings with neonatal mortality rates of 30 per 1000 live births or higher. Chlorhexidine was expected to replace the use of harmful traditional substances such as the topical application of cow dung for cord dressing [12]. Clean dry cord care was recommended by WHO for newborns delivered in health facilities. To foster a predictable availability of chlorhexidine digluconate 7.1% gel in Nigeria through domestic production, JSI-TSHIP collaborated closely with and supported the Federal Ministry of Health and the National Agency for Food and Drug Administration and Control (NAFDAC) in a process that prequalified pharmaceutical companies. As of January 2015, confirmed, executable local manufacturing capacity was estimated at 60 million tubes, well above the annual 6.9 million estimated births in 2015. Bauchi and Sokoto States added chlorhexidine digluconate 7.1% gel to their essential medicines lists as a key enabling step towards routine procurement.

To increase demand and use of chlorhexidine gel for umbilical cord care, the project supported state government officials in the establishment, reactivation and training of Ward Development Committees (WDC) and their members and Community Based Health Volunteers (CBHV); these entities forged linkages between potential clients in communities and the facilities in the nascent, statewide primary health care system. There were 2440 CBHV in Sokoto and 3230 in Bauchi States in regular interactions with women, identifying and counseling pregnant women through home visits, promoting antenatal clinic (ANC) visits, as well as promoting child health and routine immunization services. Evidence has shown that women that attended ANC clinics had a higher likelihood to deliver in a health facility [13, 14]. Ahead of its distribution of chlorhexidine gel, there were extensive consultations with communities and influential leaders including high traditional leaders, Emirs and district heads, to secure their support for the medicine and well as the distribution program. The steps and processes undertaken to secure demand for the product in Sokoto State, for example are described in detail elsewhere [15]. CBHV were trained on 20 key health behaviors, counseling techniques and recordkeeping. They were trained on the hygienic, topical application of chlorhexidine digluconate 7.1% gel to the newborn cord [15]. The barriers to the uptake of chlorhexidine digluconate 7.1% gel and how they interplay in Bauchi and Sokoto States have not been well understood. A better understanding of its uptake will inform enhanced choices in a more durable scale up of chlorhexidine digluconate 7.1% gel use.

### Objective of study

The purpose of this analysis was to examine the trends in cord care practices between 2012 and 2015, a period that coincided with the introduction and scale up of the use of chlorhexidine digluconate 7.1% gel for umbilical cord care in Bauchi and Sokoto States.

## Methods

TSHIP utilized the Lot Quality Assurance Sampling (LQAS) methodology to monitor and evaluate MNCH, family planning and reproductive health programs and interventions [16, 17]. LQAS is a small-sample alternative to traditional population-based survey methodology, which has gained increasing application in rapid turnaround public health evaluations [17, 18]. Details of this methodology have been described elsewhere [19]. It is mostly useful in determining the program coverage status of a “supervision area” (SA) against a set target or intervention coverage. Using this method, interventions, programs or SAs are classified and prioritized in the course of implementation as high priority if they achieve less than expected levels of performance (predetermined targets or benchmarks) or assigned to low priority when expected results have been achieved. On this basis, decision to shift program resources to low performing interventions or programs with potential for higher impact is easily facilitated. Estimates from the SAs are typically aggregated to obtain the coverage of an indicator in a given catchment area which in this case, is the State [20, 21]. In each round, a sample of 19 respondents from each of the sampled locations in LGAs (the SAs) provided acceptable levels of statistical error (90 to 95% confidence level, classification errors; α ≤ 5%, and β ≤20%) [22, 23] to inform decisions [21]. This sample size was based on a statistically determined decision rule (*d*), adjusted to the sample size and a predetermined coverage threshold (prevalence) [24].

These data were obtained in November 2012 in both states (for baseline), in December 2013 for Sokoto State and in March 2014 in Bauchi State (for midline) and in February 2015 for end-line (in both states). Data from three rounds of LQAS surveys were originally conducted to monitor and to ultimately evaluate impact of the TSHIP programs and the performance of the Local Government Areas (LGAs). A baseline, follow-up and an end-line LQAS survey were each conducted in November 2012, December 2013 (in Sokoto State)/March 2014 (in Bauchi State and in February 2015 respectively. In the surveys, LGA were classified as supervision areas (SAs). Identical, pre-tested questionnaires were used each time to obtain data. A rigorous, multistage random sampling method was applied in the selection of 19 LQAS locations (villages and hamlets) in each of the 20 and 23 LGAs of Bauchi and Sokoto States respectively. Subsequently, using systematic random sampling procedures, 19 households with mothers of children 23 months old and below, were selected from a compiled list of households in each of the 19 sampled locations, Data collection in each round was preceded by a nine-day long intensive, detailed-oriented training course for data collectors and field supervisors. Individual and household level data obtained through closed-ended questions, focused on mothers’ (caregivers) socio-demographic characteristics, place of last delivery, who assisted during delivery, who cut the umbilical cord, what instrument was used to cut the umbilical cord, what was applied on the cord stump and duration of application.

### Analysis

EpiData version 3.1 was utilized for electronic data capture. Descriptive analyses were conducted with purposefully preprogrammed Microsoft Excel worksheet and STATA© (Version 10). For the baseline, follow-up and end-line surveys, we estimated and compared the average coverage of each indicator for the LGAs with an acceptable level of statistical errors of α ≤ 5%, and β ≤20% [18, 22, 23, 25]. The classification of the performance of an LGA was done using a statistically determined decision rule determined by the SA sample size of 19 households and pre-determined coverage benchmarks (or targets). The LGA estimates from all the LGAs in each state were aggregated and weighted by the proportional contribution of population of each LGA, to estimate the coverage for the entire State. Consistent with LQAS methodology, confidence intervals of the estimated coverage were calculated with finite sample correction, weighting samples relative to the respective LGA population.

## Results

### Demographic characteristics

A total of 817 households—380 in Bauchi State and 437 in Sokoto State were selected in each of the three survey years. For three rounds, the sample size of households totaled 1140 in Bauchi State and 1311 Sokoto State. Table 1 describes the background information of respondent mothers that had had a live birth delivery less than 2 years prior to the surveys. The age of mothers with a recent delivery spanned the entire child bearing age range of 15–49 years. The mean age of the mothers did not differ significantly within either state across the three survey waves (Table 1). The majority of the women surveyed in both states had no formal education. However, the percentages of mothers with no formal education were lower in Bauchi State (69.5, 63.7 and 71.1%, in 2012, 2013 and 2015, respectively) than in Sokoto State (90.4, 89.7 and 87.9%). The percentages of those who attained primary education were between 16 and 23% in years of the surveys in Bauchi State and between 6 and 8% in Sokoto State. Over 90% of respondents in Bauchi State and 98% in Sokoto State, practiced Islam. There were about 6% to 9% of respondents in Bauchi State and about 1% in Sokoto State that reported that they practiced Christianity. There were no statistically significant differences in the age, education and the religion of the mothers across the survey years in both states.

**Table 1:** Background Information of Mothers and Caregivers

State:	Bauchi State			Sokoto State		
Year:	2012	2013	2015	2012	2013	2015
Total sample	380	380	380	437	437	437
Age distribution of care mothers (caregivers) (%)						
15-19 yrs	51 (13.4%)	53 (13.9%)	36 (9.5	55 (12.6%)	52 (11.9%)	41 (9.4%)
20-24 yrs	104 (27.4%)	111 (29.2%)	101 (26.6%)	96 (22.0%)	110 (25.2%)	96 (22.0%)
25-29 yrs	106 (27.9%)	84 (22.1%)	93 (24.5%)	103 (23.6%)	110 (25.2%)	116 (26.5%)
30-34 yrs	64 (16.8%)	69 (18.2%)	88 (23.2%)	104 (23.8%)	96 (22.0%)	97 (22.2%)
35-39 yrs	32 (8.4%)	34 (8.9%)	37 (9.7%)	48 (11.0%)	42 (9.6%)	42 (9.6%)
40-44 yrs	18 (4.7%)	20 (5.3%)	22 (5.8%)	25 (5.7%)	23 (5.3%)	36 (8.2%)
45-50 yrs	5 (1.3%)	9 (2.4%)	3 (20.8%)	6 (1.4%)	4 (0.9%)	9 (2.1%)
Mean age (SD)	26.9 (6.9)	26.5 (7.1)	27.0 (6.5)	27.1 (6.9)	26.8 (6.6)	30.0 (7.0)
	Pearson chi2(12) = 15.2 Pr = 0.23			Pearson chi2(12) = 10.7 Pr = 0.55		
Education attainment by year (%)						
No Education	264 (69.5%)	242 (63.7%)	270 (71.1%)	387 (90.4%)	392 (89.7%)	384 (87.9%)
Primary	77 (20.3%)	87 (22.9%)	64 (16.8%)	28 (6.5%)	25 (5.7%)	33 (7.6%)
Secondary	30 (7.9%)	46 (12.1%)	37 (9.7%)	8 (1.9%)	15 (3.4%)	14 (3.2%)
Higher	9 (2.4%)	5 (1.3%)	9 (2.4%)	5 (1.2%)	5 (1.1%)	6 (1.4%)
	Pearson chi2(6) = 9.9876 Pr = 0.125			Pearson chi2(6) = 3.5552 Pr = 0.737		
Religion pattern by year (%)						
Catholic	3 (0.8%)	7 (1.8%)	6 (1.6%)	1 (0.2%)	1 (0.2%)	0 (0.0%)
Other Christian	22 (5.8%)	29 (7.6%)	26 (6.8%)	3 (0.7%)	5 (1.1%)	3 (0.7%)
Islam	353 (92.9%)	344 (90.5%)	346 (91.1%)	430 (98.4%)	431 (98.6%)	434 (99.3%)
Traditionalist	2 (0.5%)	0 (0.0%)	2 (0.5%)	3 (0.7%)	0 (0.0%)	0 (0.0%)
	Pearson chi2(6) = 4.71 Pr = 0.581			Pearson chi2(8) = 10.42 Pr = 0.237		
Household Wealth Index						
Mean (SD)		2.0 (0.80)	1.9 (0.84)		1.9 (0.82	2.0 (0.86)

### Places where deliveries took place

**Fig. 1:**
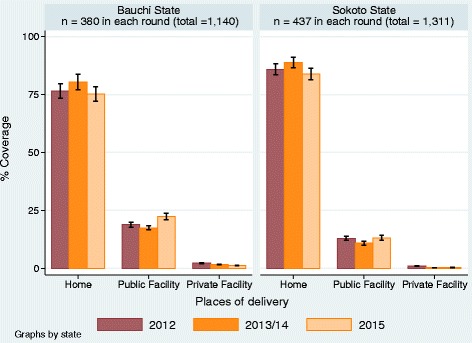
Places where Delivery Occurred. 2012–2015

Over 75% and 80% of deliveries in Bauchi and Sokoto States respectively took place at home (Fig. 1). Home deliveries rose from 76.6% (95% CI: ±3.2%) in 2012 to 80.3% (95% CI: ±3.4%) in 2013 and fell to 75.2% (95% CI: ±3.2%) in 2015 in Bauchi State. However, these changes were not statistically significant. The pattern was similar in Sokoto State where home deliveries rose slightly from 84.9% (95% CI: ±2.3%) in 2012 to 85.4% (95% CI: ±2.2%) in 2013 and fell to 83.9% (95% CI: ±2.5%) in 2015. These changes were also not statistically significant. The percentage of deliveries in public facilities was lower than home deliveries and the trend in public facility delivery was the reverse of the home delivery in both states. Public facility deliveries in Bauchi State rose significantly from 18.8% (95% CI: ±1.1%) and 17.5% (95% CI: ±0.8%) in 2012 and 2013 respectively, to 22.4% (95% CI: ±1.4%) in 2015. Similarly deliveries in public facilities rose from 12.9% (95% CI: ±0.8%) and 10.9% (95% CI: ±0.8%) in 2012 and 2013 respectively to 13.2% (95% CI: ±1.0%) in 2015 in Sokoto State. Deliveries in private facilities were negligible particularly in Sokoto State. There were statistically significant more deliveries at home and less deliveries in the public facilities in Sokoto State than in Bauchi State, consistently in all the rounds.

### Assistance during delivery

**Fig. 2:**
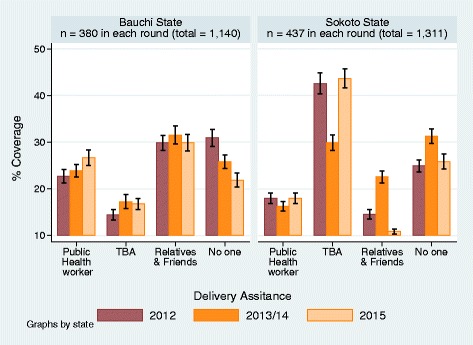
Who Assisted during Delivery?

In Bauchi State, deliveries were most frequently assisted by relatives and friends. There were no statistically significant differences in the percentage of deliveries assisted by relatives and friends across the survey periods, at 29.8% (95% CI: ±1.6%), 31.5% (95% CI: ±1.9%) and 29.9% (95% CI: ±1.8%) in 2012, 2013 and 2015 respectively (Fig. 2). Next were deliveries with no one present which declined form 30.9% (95% CI: ±1.8%) in 2012 and 25.8% (95% CI: ±1.4%) in 2013 to 21.9% (95% CI: ±1.5%) in 2015. In sharp contrast, deliveries assisted by a public health worker increased significantly from 22.7% (95% CI: ±1.4%) in 2012 to 26.6% (95% CI: ±1.7%) in 2015. Also TBA-assisted deliveries rose significantly increased from 14.4% (95% CI: ±1.2%) in 2012 to 16.8% (95% CI: ±1.2%) by 2015.

In Sokoto State, the percentage of deliveries attended by TBAs changed from 42.6% (95% CI: ±2.2) in 2012, to 29.9% (95% CI: ± 1.7%) in 2013 and then rose significantly to 43.7% (95% CI: ±2.1%) by 2015. Between 23% and 30% had no one assisting them during delivery contrasting the trend in TBA assistance. In contrast, the percentage of women delivering alone changed from 24.9% (95% CI: ±1.3%) at baseline to 31.2% (95% CI: ±1.6%) at midline and was 25.8% (95% CI: ±1.6%) at end-line. There was no statistically significant difference from baseline to end-line although the increase from baseline to midline and the decrease from mid-line to end-line were statistically significant. With regards to relatives and friends, the mid period (2013) increase in the assistance they provided to women during delivery of 22.5% (95% CI: ±1.3%) coincided with the 2013 dip observed in the assistance provided by the TBAs. About 18.0% (95% CI: ±1.1%), 16.3% (95% CI: 1.0%) and 18.0% (95% CI: ±1.1%) in 2012, 2013 and 2015 respectively, were assisted by public health workers. This was slightly higher than the proportion that delivered in public health facilities which ranged from 11% to 13%.

Broadly, delivering alone with no assistance decreased in both States while deliveries assisted by public health workers increased between 2012 and 2015. This trend was more pronounced in Bauchi State. In reality, TBAs were often also relatives and friends and vice versa. As such, the distinction between these two categories of delivery attendants in this data is less clear. Consequently, upwards of 50% of deliveries in Bauchi and more than 80% in Sokoto State were assisted by TBAs, relatives and/or friends. Deliveries that were assisted by public health workers and relatives and friends rose by a statistically significant greater proportion in Bauchi State than in Sokoto State in all the surveys. Whereas, TBA assisted deliveries were a significantly greater proportion in Sokoto State than in Bauchi State.

### Who cut the cord after delivery?

**Fig. 3:**
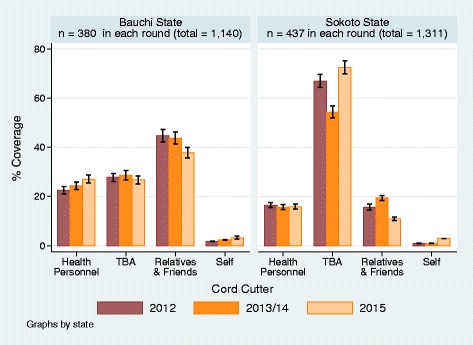
Who Cut the Umbilical Cord?

The trend in “who cut the cord after delivery” generally reflected changes in the trend in which assistants had attended the deliveries in both states (Fig. 3). However, the percentages of women that cut the cord themselves were strikingly lower than the percentage of women who delivered alone. In both state, between 22% and 31% across the survey years delivered alone and unassisted. The women cut the cord by themselves in less than 3% of all deliveries. Observations from the field suggested that it was common for women who had delivered at home to invite a TBA to “manage” the third stage of labor and cut the umbilical cord after the babies had been born. This may explain why there were more deliveries that had the umbilical cords cut by TBAs than there were TBAs assisted deliveries. As TBAs, relatives and friends were akin to the places and times of delivery and provided most of the delivery assistance, they have also mostly managed the cord care processes. TBAs and relatives and friends as a group were responsible for cutting the cord of the newborn in close to 70% of deliveries in Bauchi State and over 80% in Sokoto State. In Bauchi State, the TBAs participation in cutting the cord decreased significantly from 27.7% (95% CI: ±1.7%) and 28.6% (95% CI: ±1.9%) in 2012 and 2013 respectively to 26.7% (95% CI: ±1.6%) in 2015. In Sokoto State, TBAs’ role increased significantly from 66.9% (95% CI: ±2.7%) and 54.3% (95% CI; ±2.4) in 2012 and 2013 to 72.5% (95% CI: ±2.6%) by 2015. The trend in the percentage of deliveries that had health personnel cutting the cord was congruent with the percentage of women who were assisted by health personnel (Figs. 2 and 3). This percentage was about 25% in Bauchi State, with a statistically insignificant increase across the years and less than 20% in Sokoto State; where there was a statistically insignificant slight decrease.

### What instrument was used to cut the umbilical cord?

**Fig. 4:**
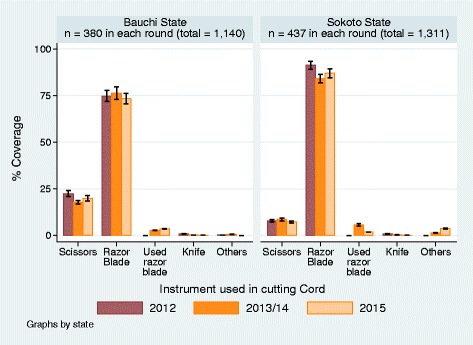
Instruments used in cutting the Umbilical Cord

New razor blades were used to cut the cord in about 75% of the deliveries in Bauchi and in over 80% in Sokoto States in the pooled data. Scissors were used less frequently, being used between 17% and 23% in Bauchi and 7% and 9% in Sokoto States (Fig. 4). Other instruments that were less frequently used included, pre-used razor blades, knives, and other unspecified instruments. The trend generally reflected the trends in who assisted and who cut the cord during deliveries. While surgical scissors are mainly available and used in public health facilities, new razor blades were traditionally used by the TBAs, friends and relatives assisting during delivery. It was observed that razor blades were infrequently included in the delivery list of mothers that delivered in public health facilities. Notwithstanding the similarities in the pattern in the instrument used for cutting the cord between Sokoto and Bauchi States, razor blade was used in Sokoto State significantly more frequently than in Bauchi State. Scissors were used to cut the cord at a significantly higher frequency in Bauchi State than in Sokoto State. The pattern of instrument use is indicative of where deliveries took place and the person who cut the umbilical cord after delivery.

### Materials used to dress umbilical cord stumps

**Fig. 5:**
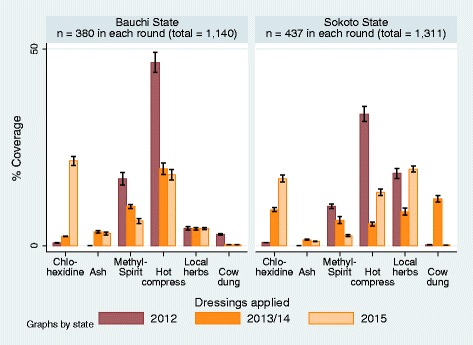
Trends in Type of Dressing Materials used for Cord Dressing 2012–2015

Chlorhexidine, ash, methylated spirit, hot compress, local herbs, and cow dung were the most commonly reported cord care products topically applied to dress freshly-cut umbilical stumps (Fig. 5). Hot compress and local herbs were used in cord dressing in the majority of the deliveries. These methods together with ash and cow dung were likely to be used by TBAs, friends and relatives who mostly attended the deliveries and managed the cord stump. In Bauchi State, the application of hot compress as a cord dressing dropped significantly from 46.5% (95% CI: ±2.6) in 2012 to 19.5% (95% CI: ±1.4%) and 18.0% (95% CI: ±1.3%) in 2013 and 2015 respectively. The percentage of women who reported the application of local herbs as cord dressing remained unchanged at 4% from 2012 to 2015, while the application of ash rose slightly from 0% in 2012 to 3.5 (95% CI: ±0.3) and 3.1% (95% CI: ±0.3) in 2013 and 2015 respectively. The application of cow dung for cord dressing fell from 2.9% in 2012 to 0.3% in 2013 and 2015. In Sokoto State, hot compress application significantly fell from 33.4% (95% CI: ±1.9%) in 2012 to 13.5% (95% CI: ±0.8%) in 2015. Local herb application remained largely unchanged between 2012 and 2015. Cow dung application was about 0.3% in 2012 and 2015 except for a spike of about 12% in 2013. The application of ash rose from 0% 2012 to 1.5% and 1.1% in 2013 and 2014 respectively.

The data trends indicated that the application of methylated spirits was being replaced with chlorhexidine gel as the agent of preference for newborn cord care in both States. A statistically significant greater proportion of cord was dressed with chlorhexidine dressing however, in Bauchi State than in Sokoto State in the third round of the survey. In Bauchi State, the application of chlorhexidine gel rose sharply from 0.7% in 2012 and 2.4% (95% CI: ±0.1%) in 2013 to 21.5% (95% CI: ±1.1%) in 2015 in contrast to methylated spirit which decreased from 17.0% (95% CI: ±1.6%) in 2012 progressively to 9.9% (95% CI: ±0.5%) and 6.3% (95% CI: ±0.6%) in 2015. Similarly, in Sokoto State, the use of chlorhexidine gel for cord dressing rose from 0.8% (95% CI: ±0.1%) in 2012 to 9.2%% (95% CI: ±0.5%) in 2013 and 17.1% % (95% CI: ±0.8%) in 2015. In contrast, the use of methylated spirits for cord dressing dropped from 10.0% (95% CI: ±0.6%) in 2012, 6.5 (95% CI: ±0.8%) in 2013 to 2.6% (95% CI: ±0.2%) in 2015. The proportion of women who reported that chlorhexidine was used for cord dressing in their last delivery was similar to the proportion that reported their delivery took place in a public health facility and was assisted by a public health worker. Chlorhexidine may be replacing the use of methylated spirit for cord dressing in public health facilities. TBAs typically use hot compress and local herbs for cord dressing and these choices are steeped in the local traditional practice. All in all, while the use of chlorhexidine gel increased between 2012 and 2015, the percentage of women that reported the use of hot compress and/or local herbs for cord dressing remained largely unchanged over the same period, and the use of cow dung fell sharply. Although the use of methylated spirit and the hot compress decreased significantly in Bauchi state, they were used for cord dressing in significantly greater proportions of deliveries in Bauchi state than in Sokoto State.

## Discussion

Our results highlight the ubiquitous role of TBAs as arbiters of the cultural tradition in umbilical cord practices in Bauchi and Sokoto States wherein at least three quarters of all deliveries occurred at home. This role is exerted in a context in which nearly half of deliveries in Bauchi State and four-fifths of those Sokoto States were assisted by TBAs, relatives or friends, which held steady through the study period. TBAs are also as likely to be friends or relatives of mothers in Bauchi and Sokoto State. As such, these two categories may not be as distinct as previously thought in this setting. While the percentage of deliveries by relatives and friends and TBA’s remained unchanged across the years in Bauchi state, the trend in the changes suggest inverse correlation to further indicate the notion that TBAs and relatives and friends who mainly assisted in deliveries were more often the same persons. Deliveries with the women alone with no assistance decreased significantly by 2015 to between 22% and 25% in both States. The trends over the years mirror the pattern in deliveries assisted by public health workers which significantly increased to between 18% in Sokoto and 28% in Bauchi States in 2015. Facility-based delivery (Facilities remain the safest place of birth), stood at one in five deliveries in Bauchi State and one in eight in Sokoto State. The trend in “who cut the cord after delivery” was generally linked to which assistant attended the deliveries. However, the percentages of women who cut the cord themselves were strikingly lower than the percentage of women who delivered alone (contrasting Figs. 2 and 3). Women, who delivered at home alone with no one assisting, most likely invited TBAs to cut the cord in the third stage of labor because much more TBAs cut the cord than they assisted in deliveries in the second stage of labor. Consequentially, in over 75% in Bauchi and over 80% in Sokoto States, new razor blades were used for cutting the newborn cord in contrast to the much lower percentages of the use of scissors which were mainly used in the public health facilities. As new razor blade was generally and conveniently used by the TBAs, these findings underscore the pervasive immediate postpartum role of TBAs.

The use of chlorhexidine gel for cord dressing significantly increased in coverage to the highest in 2015 in both States, coinciding with decreasing application of methylated spirits for cord care. Methylated spirit, which was readily available for general antisepsis in public health facilities, was being replaced with chlorhexidine. Hot compress, local herbs, ash and cow dung which were mainly used to dress severed cords by TBAs, relations and friends maintained about the same trend and coverage as those of “who cut the cord” and “who assisted during the deliveries” (Figs. 3 and 4). Unlike the substitution of methylated spirit with chlorhexidine by the public health workers, there were little or no change in cord care practice among the TBAs. The use of ash, local herbs and hot compress remained prevalent, although use of cow dung declined somewhat over the years to 2015.

Our findings have direct implications for future policy and interventions for the scaled up distribution and use of chlorhexidine gel to prevent cord infection, especially in the culturally homogeneous northern regions of Nigeria. Although TBAs which were incorporated into the Nigerian health system in the ‘80s and ‘90s to advance the Bamako initiative Primary Health Care systems have been phased out as they are regarded as unskilled, they have nonetheless remained highly relevant for deliveries in the communities. As strong custodians of cultural birth traditions, TBAs poorly adapted to modern cord practices and continued to perpetrate and adhere to unwholesome cord care practices. To avoid the continued adverse impact of TBA activities on the reduction of cord infection, there must either be a conclusive phasing out of the TBAs as World Health Organization has recommended to countries or a retraining to refocus their activities toward appropriate cord care practices. TBAs role in the healthcare systems can and should actively be readapted to play an important role in increasing access to health care and services effectively linking the community and the formal health system similarly to the role commonly played by community based health volunteers in Bauchi and Sokoto States [26]. Although a study in Somalia explored the feasibility of changing the roles of TBAs to that of birth companions and promoter of skilled birth attendance, [27] evidence remains insufficient to establish the potential of TBA training to improve perinatal mortality [28, 29]. However, this study showed that with the appropriate incentives, TBAs readily embraced and adopted their new roles of referring women to health facility at the emergence of complications, instead of conducting home deliveries. The TBAs accompanied or referred mothers to facilities for deliveries, prenatal or postnatal care. A CBHV-trained TBA tag-teaming could be a feasible strategy to explore as has been demonstrated in a study from Zambia [30].

### Limitations

The samples for this study consisted of mothers or caregivers of 0 to 23 months old children. Recall bias was therefore likely, particularly from responders (mothers) whose birth experiences were longer ago. However, as deliveries represent profound experiences to women particularly in this region, recall over a considerable length of time may in fact be consistent and reliable. There were no birth records for the majority of the children as over 75% of deliveries took place at home. It appeared that responders had no perceptible difficulties in recalling events around the birth of the last child in the interviews.

## Conclusions

In conclusion, unwholesome umbilical cord care practices remained prevalent in Bauchi and Sokoto States of Nigeria. Although the recent introduction and the scaling up of the use of chlorhexidine gel positively changed the cord care practices toward safer practices by providers in public health facilities, TBAs, friends and relatives played the strongest immediate postpartum roles. Unhealthy cord care practices such as use of ash, cow dung and hot compress for cord dressings remained present in the communities. To mitigate the influence of TBAs on deliveries, policy and program efforts need to be intensified to accelerate the rising proportion of births attended by a skilled birth attendant. In tandem, both States should explore and adopt the strategies for retraining and refocusing the existing TBAs to forge stronger links between the communities and the primary healthcare facilities similarly to the roles played by the CBHVs in the communities.

## Abbreviations

CBHV: Community Based Health Volunteer
FUTURES: Futures Group
JSI: John Snow Inc. Research and Training
LGA: Local Government Authority
LQAS: Lot quality assurance sampling
MDG: Millennium Development Goals
MNCH: Maternal Neonatal and Child Health
NAFDAC: National Agency for Food and Drug Administration and Control
NMR: Neonatal mortality rate
SA: Supervision area
SD: Standard deviation
TBA: Traditional birth attendant
TSHIP: Targeted States High Impact Project
WDC: Ward Development Committee
WHO: World Health Organization
